# p38 MAPK mediates glial P2X7R-neuronal P2Y1R inhibitory control of P2X3R expression in dorsal root ganglion neurons

**DOI:** 10.1186/s12990-015-0073-7

**Published:** 2015-11-05

**Authors:** Yong Chen, Guangwen Li, Li-Yen Mae Huang

**Affiliations:** Department of Neuroscience and Cell Biology, University of Texas Medical Branch, Galveston, TX 77555-1069 USA

**Keywords:** Dorsal root ganglion, P2X3, P2X7, P2Y1, p38, Pain, Purinergic, Satellite glial cell

## Abstract

**Background:**

We have previously shown that endogenously active purinergic P2X7 receptors (P2X7Rs) in satellite glial cells of dorsal root ganglia (DRGs) stimulate ATP release. The ATP activates P2Y1Rs located in the enwrapped neuronal somata, resulting in down-regulation of P2X3Rs. This P2X7R-P2Y1-P2X3R inhibitory control significantly reduces P2X3R-mediated nociceptive responses. The underlying mechanism by which the activation of P2Y1Rs inhibits the expression of P2X3Rs remains unexplored.

**Results:**

Examining the effect of the activation of p38 mitogen-activated protein kinase on the expression of P2X3Rs in DRGs, we found that the p38 activator, anisomycin (Anis), reduced the expression of P2X3Rs. Blocking the activity of SGCs by the glial Krebs cycle inhibitor, fluorocitrate, did not change the effect of Anis. These results suggest that neuronal p38 plays a major role in the inhibition of P2X3R expression. Western blotting analyses showed that inhibiting P2Y1Rs by MRS2179 (MRS) or blocking P2X7Rs by either oxATP or A740003 reduced pp38 and increased P2X3R expression in DRGs. These results are further supported by the immunohistochemical study showing that P2X7R and P2Y1R antagonists reduce the percentage of pp38-positive neurons. These observations suggest that activation of P2X7Rs and P2Y1Rs promotes p38 activity to exert inhibitory control on P2X3R expression. Since activation of p38 by Anis in the presence of either A740003 or MRS could overcome the block of P2X7R-P2Y1R inhibitory control, p38 in DRG neurons is downstream of P2Y1Rs. In addition, inhibition of p38 by SB202190 was found to prevent the P2X7R and P2Y1R block of P2X3R expression and increase P2X3R-mediated nociceptive flinch behaviors.

**Conclusions:**

p38 in DRG neurons downstream of P2Y1R is necessary and sufficient for the P2X7R-P2Y1R inhibitory control of P2X3R expression.

## Background

DRG neurons are primary sensory neurons which convey somatic and visceral sensory information, including pain sensation, from peripheral tissues to the spinal cord. The cell bodies (somata) of DRG neurons are tightly packed in the ganglion [1]. The unique morphological features of DRG neurons are that the somata of DRG neurons lack dendrites and do not form synaptic connections with each other [2]. Instead, each soma is tightly wrapped by a layer of SGCs. The soma-SGC unit is enclosed by a connective tissue sheath to form a structural unit [1, 3]. This structural arrangement between neuronal soma and SGCs suggests that soma-SGC-soma communication is essential for determining the somatic activity of DRG neurons.

The robust release of ATP from the somata of DRG neurons [4] and the abundant expression of purinergic receptors in neurons and SGC cells in DRGs and trigeminal ganglia suggest that purinergic signaling is a major means used by neuronal somata and SGCs in sensory ganglia to communicate with each other [5–12]. Among the seven ionotropic purginergic P2XR subtypes (P2X1-P2X7R), P2X3R is the most abundantly expressed subtype in DRG neurons and plays a key role in the transmission of nociceptive information [10, 13–17]. In contrast, P2X7R is the most abundantly expressed P2XR subtype in SGCs of DRGs [4, 18–20]. Activation of P2X7Rs has been implicated in apoptosis, maturation and release of cytokines, regulation of receptor trafficking and production and maintenance of pain in the brain and spinal cord [21–24]. We and others have shown that the P2X7R expression is not detectable in neurons and P2X3Rs are not found in SGCs of DRGs [4, 18–20]. This exclusive expression of P2X3R in neurons and P2X7R in SGCs facilitates our determination of the interaction between glial P2X7Rs and neural P2X3Rs. We showed previously that P2X7Rs in SGCs are endogenously active and mediate a significant portion of the basal ATP release in the DRG [10, 20]. The released ATP activates the metabotropic P2Y1Rs in the somata of DRG neurons and reduces the expression of P2X3Rs. This P2X7R-P2Y1R-P2X3R control exists in both adult and immature rats [20, 25]. The mechanism by which the activation of P2Y1Rs leads to the reduction of P2X3R expression has yet to be elucidated.

p38, together with ERK and JNK, is a major member of MAPKs [26]. In response to signals or stress, p38 MAPK, a serine-threonine kinase, is phosphorylated and activated to regulate cytosolic and nuclear targets [27, 28]. Inflammation and nerve injury were found to upregulate phosphorylated p38 (pp38) in both neuronal somata and SGCs of primary sensory ganglia [29] and in spinal microglia [28, 30, 31]. Activation of purinergic receptors, P2X4Rs [32], P2X7Rs [33] and P2Y12 [34] in microglia of the spinal cord and P2Y1R in DRG neurons and peripheral terminals [35] have been shown to upregulate pp38, which led to thermal or mechanical hypersensitivity. Here we show that in contrast to the pain-producing effects of p38, activation of neuronal p38 also plays a critical role in P2Y1R-mediated inhibitory control of P2X3R expression in DRG neurons.

## Results

### Activation of p38 reduces the expression of P2X3Rs in DRG neurons

**Fig. 1:**
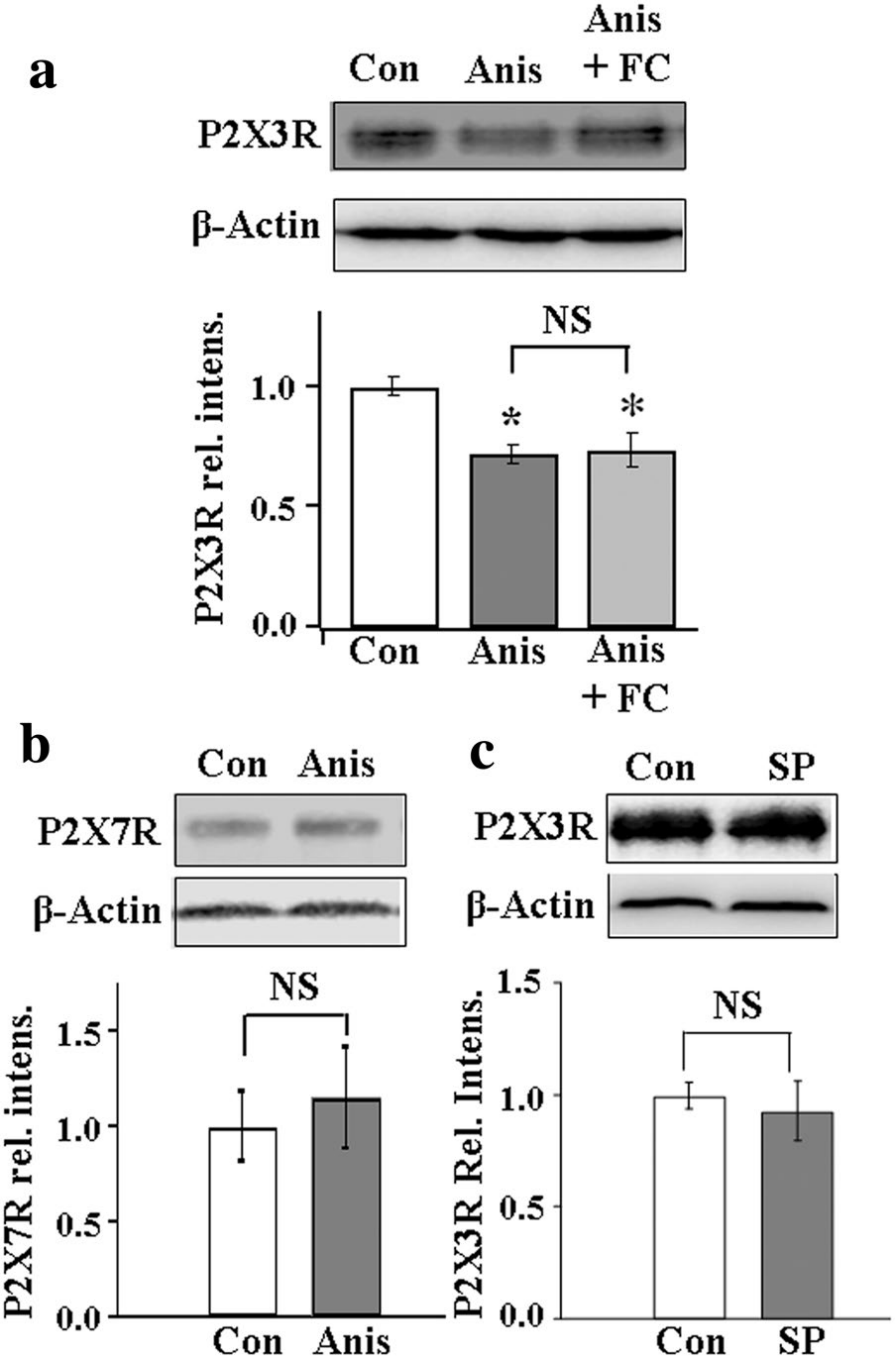
Activation of neuronal p38 reduces the expression of P2X3R. **a** Pre-treatment of DRGs with Anis (10 nM, 1 h) significantly reduced the P2X3R expression in DRGs [Anis/Con, 0.72 ± 0.04, n(Con) = 5, n(Anis) = 8]. Treating DRGs with both FC (100 µM) and Anis did not alter the inhibitory action of Anis on P2X3R expression [(Anis + FC)/Con = 0.74 ± 0.07 (mean ± SEM), n(Con) = 5, n(Anis + FC) = 6]. **b** Pre-treatment of DRGs with Anis (10 nM, 1 h) did not alter the P2X7R expression [Anis/Con, 1.15 ± 0.05, n(Con) = 4, n(Anis) = 3)]. **c** Treating DRGs with the JNK inhibitor, SP600125 (SP) (2 µM) did not change the P2X3R expression [SP/Con = 0.93 ± 0.13 n(Con) = 4, n(SP) = 3]. *P < 0.05 compared with Con; *NS* not significant, P > 0.05

We showed, in a previous study, that P2X3R expression in DRG neurons is regulated by P2X7R-P2Y1R inhibitory control [20]. To determine if p38 is involved in the P2X7R-P2Y1R-P2X3R pathway, we examined the effect of p38 activation on P2X3R expression. DRGs were acutely removed from rats and incubated with the p38 activator, anisomycin (Anis) (10 nM), for 1 h. The expression of P2X3Rs in DRGs, examined with Western analyses, was significantly reduced (Fig. 1a).

Since p38 is expressed in both neurons and SGCs in DRGs [29], it is of interest to determine if neuronal- and/or glial p38 participate in the regulation of P2X3Rs. The effect of Anis on P2X3R expression in the presence of the glia Krebs cycle inhibitor, fluorocitrate (FC), which disrupts the function of SGCs was studied. We found that Anis reduced P2X3R expression to a similar extent with and without FC (Fig. 1a). This observation suggests that the activation of “neuronal” p38 is sufficient to inhibit P2X3R expression.

Anis is known to also inhibit protein synthesis [36]. To eliminate this possibility, we examined the effect of Anis on the expression of another puringeric receptor, i.e., the P2X7R. In contrast to P2X3Rs, Anis had no effect on the expression of P2X7Rs (Fig. 1b). Thus, Anis, at 10 nM (=0.265 ng/ml), does not affect the protein synthesis of purinergic receptors. In addition, Anis can also activate JNKs to affect cell activity [37]. To determine if Anis block of P2X3R expression can be attributable to JNKs activation, the effects of Anis on P2X3R expression with and without pretreatment with the JNKs inhibitor, SP600125 (2 µM), were compared. SP600125 had no effect on P2X3R expression (Fig. 1c). These results strongly suggest that Anis reduces P2X3R expression through its activation of p38.

**Fig. 2:**
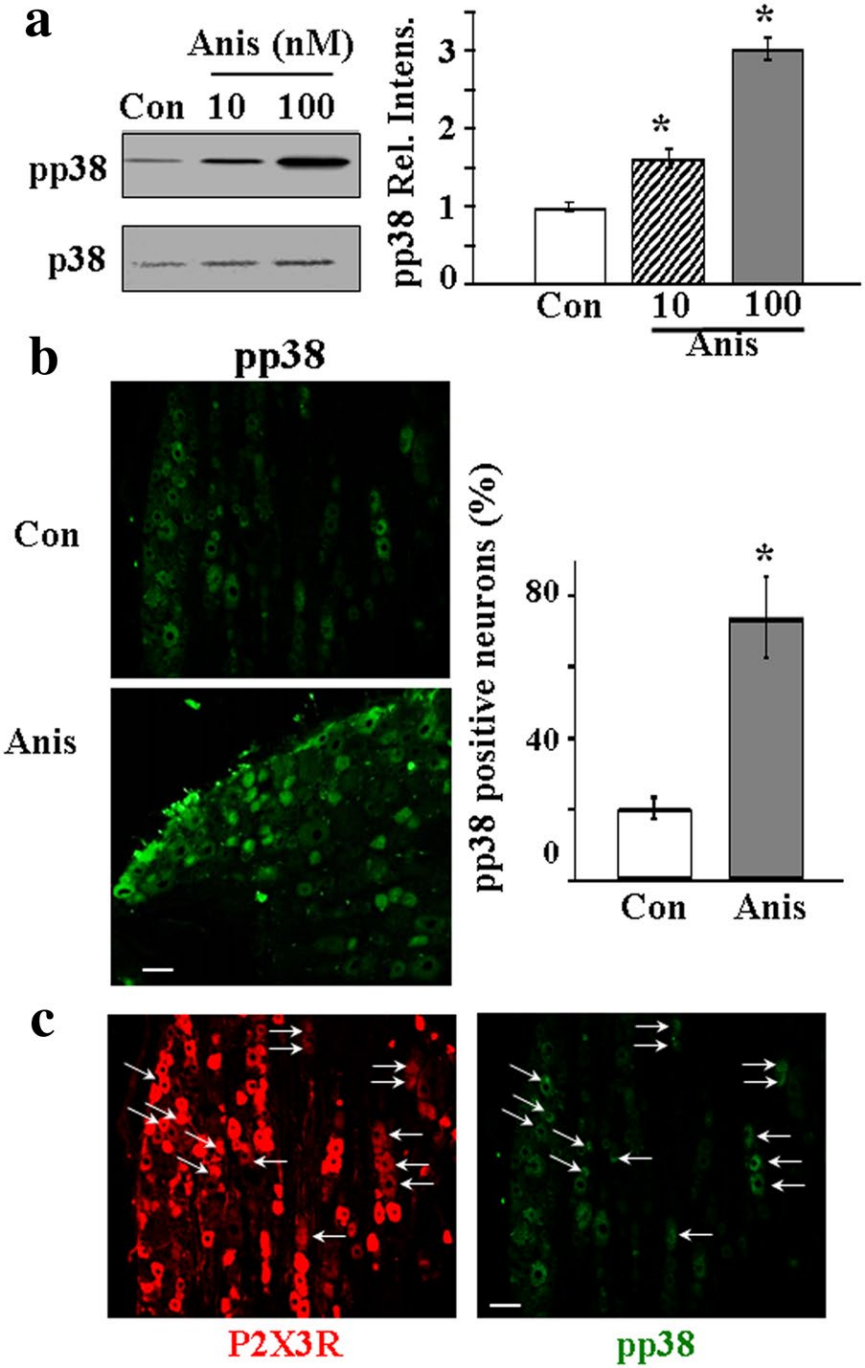
Activation of pp38 by Anis in DRGs. **a** Pre-treatment of DRGs with Anis increased the expression of pp38 in a dose-dependent manner [Anis (10 nM)/Con, 1.64 ± 0.13, n = 4; Anis (100 nM)/Con, 3.06 ± 0.20, n = 4). **b** Intrathecal application of Anis (10 nM) significantly increased the percentage of pp38 positive neurons in DRGs. Con, Anis: labeled DRG cells with the pp38 antibody in control and Anis treated DRG slices. Percentages of pp38 positive neurons were 20.4 ± 3.1 % (Control) and 74.0 ± 11.4 % (Anis). **c** Double labeled DRG neurons with pp38 and P2X3R antibodies. About 20 % P2X3R positive cells expressed pp38. *Arrows* cells labeled with both P2X3R and pp38. *Calibration bar* 50 µm, *P < 0.05 compared with Con

To further confirm that Anis treatment indeed activates pp38 in DRGs, we examined the pp38 expression using Western blotting analyses. Anis increased pp38 expression in a dose dependent manner (Fig. 2a). We then used immunohistochemical (IHC) experiments to determine the distribution of pp38 in DRG neurons. pp38 was found in ~20 % of DRG neurons in control rats (Fig. 2b). After Anis (10 nM) treatment, a majority of neurons (~74 %) expressed pp38.

Colocalization of pp38 and P2X3Rs in DRG neurons was also determined. We labeled neurons with both pp38 and P2X3R antibodies and found that ~20 % P2X3R positive neurons expressed pp38; ~33 % of pp38-containing cells were P2X3R-positive (Fig. 2c).

### p38 is involved in the P2X7R-P2Y1R inhibitory control of P2X3Rs

**Fig. 3:**
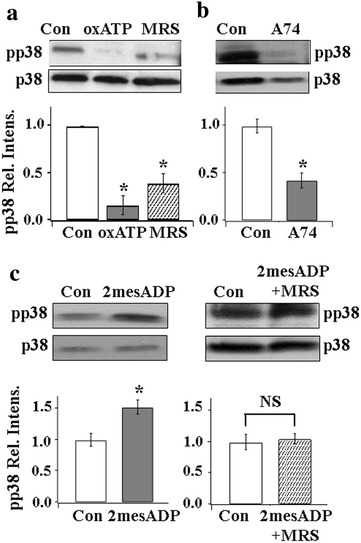
P2X7R and P2Y1R regulate pp38 expression. **a** Inhibition of P2X7R or P2Y1R activity reduces pp38 expression. Treating DRGs with the P2X7R antagonist, oxATP (100 µM), or with the P2Y1R antagonist, MRS2179 (60 µM) for 3 h greatly diminished the expression of pp38 (oxATP/Con, 0.16 ± 0.10; MRS/Con, 0.39 ± 0.10, n = 3, *P < 0.05). **b** Incubation of DRGs with the specific P2X7R antagonist A740003 (A74) also decreased the pp38 expression [(A740003/Con, 0.42 ± 0.08, n (Con) = 9, n(740003) = 7, *P < 0.05]. **c** (*Left*) Treating DRGs with the P2YR agonist, 2mesADP (100 µM), increased the pp38 expression (2mesADP/Con = 1.53 ± 0.11,*P < 0.05). (*right*) Incubation of the P2Y1R antagonist, MRS, with 2mesADP blocked the enhancing effect of 2mesADP on pp38 expression (Con/2mesADP + MRS, 1.05 ± 0.08, n = 3, NS, P > 0.05)

In our previous studies, we showed that endogenously activated P2X7Rs in SGCs evoke ATP release from satellite cells to activate P2Y1Rs in neurons and P2X7R-P2Y1R exerts inhibitory control of P2X3R expression in DRGs [20]. To determine the role of p38 in the P2X7R → P2Y1R → P2X3R pathway, the pp38 expression was determined in the presence of the P2X7R antagonist, oxATP, or the P2Y1R antagonist, MRS2179 (MRS). Both oxATP and MRS inhibited the level of pp38 (Fig. 3a), suggesting that p38 activity is regulated by P2X7Rs and P2Y1Rs. In another set of experiments, we used a specific P2X7R antagonist A740003 to inhibit P2X7R and found that pp38 expression was similarly inhibited (Fig. 3b), thus confirming that P2X7R promotes the expression of pp38. We then used the P2Y1 agonist, 2mesADP [38], to further examine the participation of P2Y1Rs in the control of p38 expression. 2mesADP was found to increase the expression pp38 (Fig. 3c, left). Because 2mesADP is known to activate P2Y1, P2Y12 and P2Y13 receptors [39], we determined if the observed action of 2mesADP is mediated by P2Y1Rs. When DRGs were treated with both 2mesADP and the P2Y1R antagonist MRS, 2mesADP no longer altered pp38 expression (Fig. 3c). Thus, the observed 2mesADP effect on pp38 is mediated by P2Y1Rs. These experiments confirmed the involvement of P2X7 and P2Y1R in promoting p38 activity in DRGs.

**Fig. 4:**
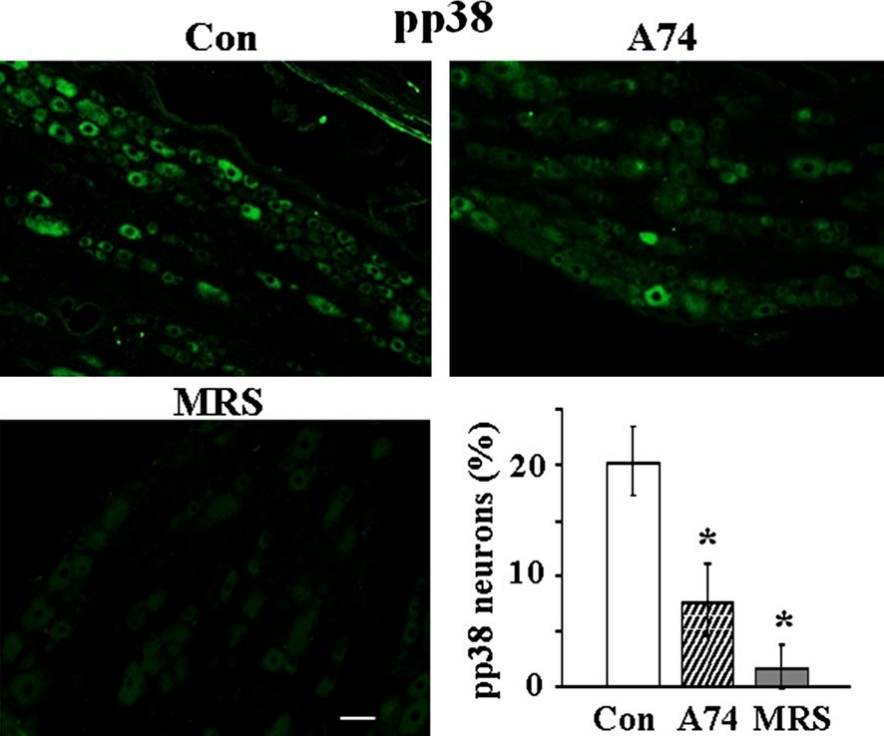
IHC evidence of the involvement of P2X7R and P2Y1R in the regulation of pp38 expression. Intrathecal applications of the P2X7R antagonist, A740003 (A74) (250 nM) or the P2Y1R antagonist MRS (60 µM) at 10 µl, twice a day for 3 days, significantly inhibited the percentage of pp38 positive neurons in DRGs (Control: 20.40 ± 3.10 %, A740003: 8.00 ± 3.33 % and MRS: 1.93 ± 1.93 %) *calibration bar*: 50 µm, n = 3, *P < 0.05 compared with control

In addition, IHC analyses were used to determine the effect of P2X7R antagonist, A74003 or the effect of the P2Y1R antagonist, MRS, on the percentage of pp38-positive cells expressed in DRG neurons. Both antagonists significantly decreased the number of pp38 positive neurons (Fig. 4), further confirming the involvement of the P2X7R and P2Y1R in promoting the p38 activity in DRG neurons.

### p38 is downstream of P2X7R-P2Y1R inhibitory control

**Fig. 5:**
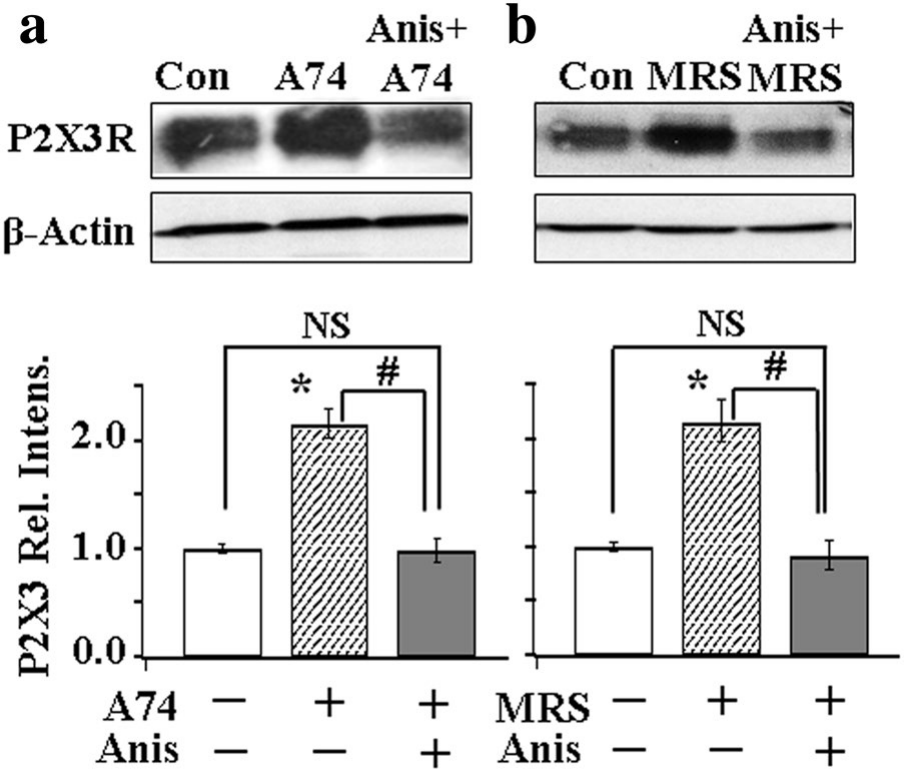
p38 is downstream of P2X7R-P2Y1R control of P2X3R expression. **a** Blocking P2X7R activation with the P2X7R antagonist, A740003 (A74) (250 nM), caused an increase in the P2X3R expression in DRGs [A74/Con, 2.17 ± 0.13, n(Con) = 14, n (A74) = 9]. Activating p38 with Anis (10 nM) overrode the enhancing effect of A74 on P2X3R expression [(Anis + A74)/Con = 0.98 ± 0.11, n(Con) = 14, n (Anis + A74003) = 9]. **b** Blocking P2Y1R activation with MRS increased P2X3R expression [MRS/Con, 2.17 ± 0.19, n(Con) = 14, n(MRS) = 9]. In the presence of Anis, MRS no longer augmented the P2X3R expression [(Anis + MRS)/Con = 0.93 ± 0.14, n(Con) = 14, n(Anis + MRS) = 8]. NS: P > 0.05; *P < 0.05 compared with [A74 (−) + Anis (−)] (**a**) or with [MRS (−) + Anis (−)] (**b**); ^#^P < 0.05 compared with [A74 (+) + Anis (+)] (**a**) or with [MRS (+) + Anis (+)] (**b**)

We then determined if p38 can control the P2X3R expression in the absence of P2X7R or P2Y1R activation. Blocking P2X7R activation by the P2X7R antagonist, A740003 was found to enhance P2X3R expression in DRGs. Studying the effect of the p38 activator, Anis, in the presence of A740003, we found that Anis could overcome A740003-induced enhancement of P2X3R expression (Fig. 5, left). Similarly, using the P2Y1R antagonist, MRS, to block P2Y1R activation, P2X3R expression was also increased (Fig. 5, right). Again, Anis blocked the MRS-induced increase in the expression of P2X3R. These observations suggest that p38 is located downstream of the P2X7R-P2Y1R pathway to control the P2X3R expression in DRG neurons.

### Activation of p38 in neurons is necessary for P2X7R-P2Y1R inhibitory control of P2X3R expression

**Fig. 6:**
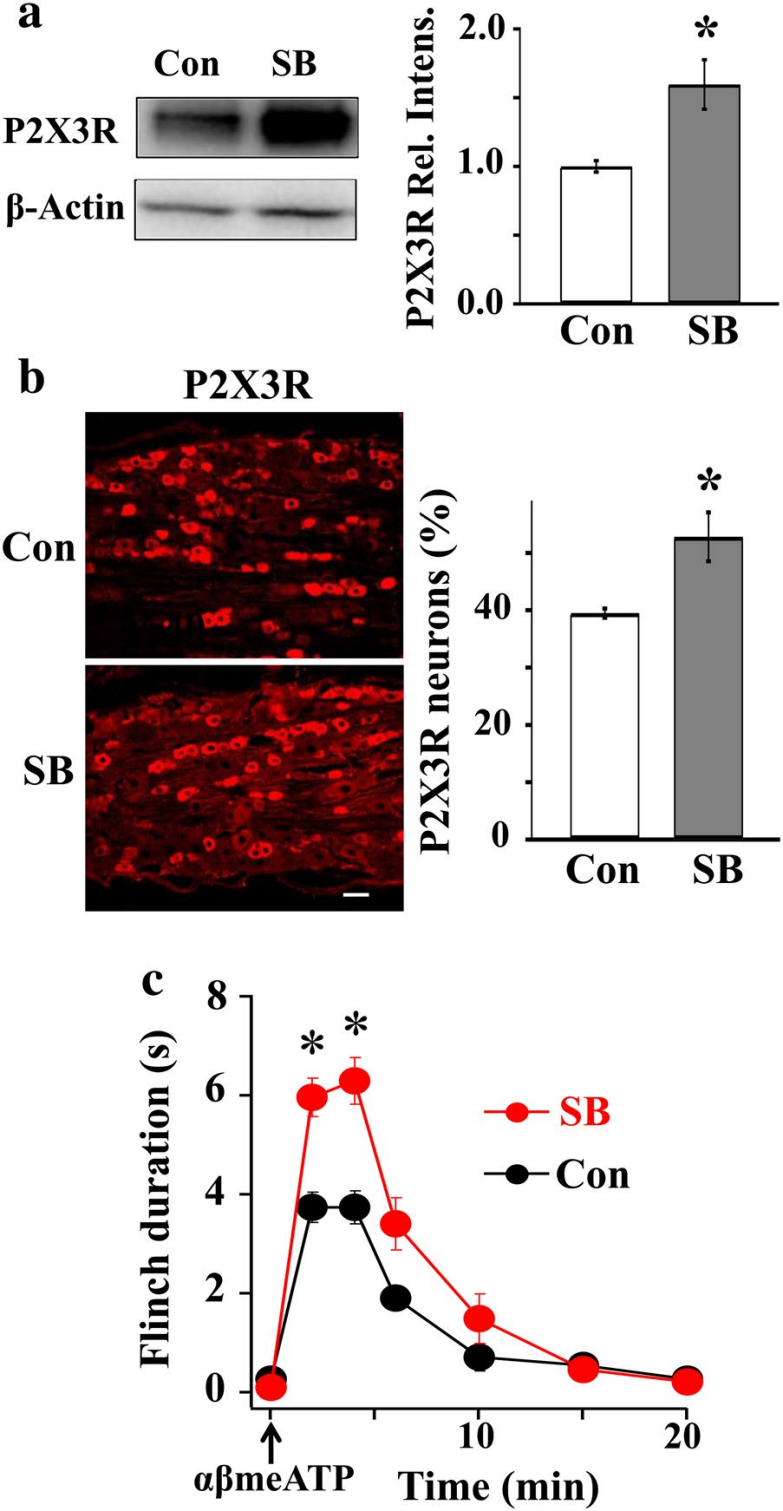
Activation of p38 in neurons is necessary for the P2X7R-P2Y1R inhibitory control of P2X3R expression. **a** p38 activation is required for P2X7-P2Y1R inhibitory control of P2X3Rs. P2X3R expression was enhanced when p38 activity was inhibited by SB (100 µM, 3 h) (SB/Con, 1.60 ± 0.14, n(Con) = 8, n(SB) = 7, *P < 0.05) **b** Intrathecal application of SB (100 µM) increased the  % of P2X3R positive neurons (Control: 39.5 ± 0.8 %, SB: 52.8 ± 4.3 %. n = 3) *P < 0.05, *bar* = 50 µm. **c** In SB treated rats, α,β-meATP induces exaggerated flinch behavioral responses in the absence of activation of p38 (n = 4) (ANOVA, *P < 0.05)

We next examined if P2X7R-P2Y1R inhibitory control of P2X3Rs can exist in the absence of p38. Freshly removed DRGs were incubated with the p38 inhibitor, SB, for 3 h and P2X3R expression was then probed. We found that SB enhanced the expression of P2X3Rs (Fig. 6a). In addition, treating DRGs with SB significantly increased the number of P2X3R positive neurons in DRG neurons (Fig. 6b). These results suggest that without p38 activation, P2X7-P2Y1 could not exert its inhibitory control on the P2X3R expression.

**Fig. 7:**
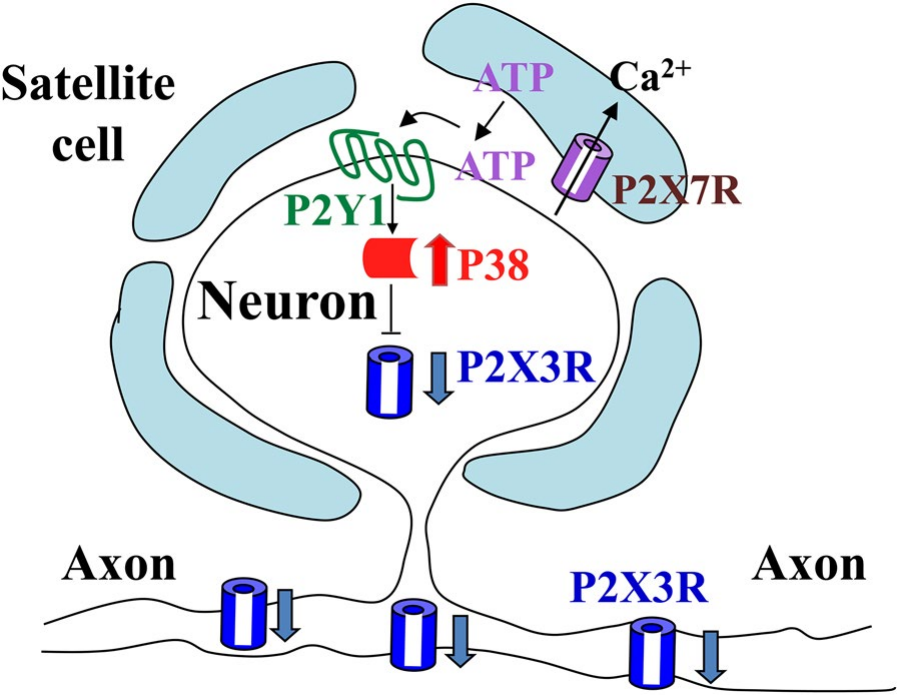
A schematic shows the involvement of p38 in P2X7R-P2Y1R-P2X3R inhibitory control. Activation of P2X7R-P2Y1R leads to the activation of p38 in DRG neurons. p38 down-regulates P2X3Rs and results in reduction of nociception

The functional consequence of p38 activation in the P2X7R-P2X3R inhibitory control of P2X3R expression was also determined. Following the synthesis of P2X3Rs in the somata of DRGs, the receptors are transported to peripheral and central nerve terminals for pain signaling. An intradermal hindpaw injection of the P2XR agonist, αβ-meATP, elicited the rat to lift its injected paw repetitively (flinching) (Fig. 6c). This nocifensive behavior is a result of the activation of P2X3Rs [17, 20, 40]. Intrathecal injections of the p38 inhibitor, SB, induced exaggerated nociceptive flinching responses (Fig. 6c). Thus, the inhibition of p38 eliminates P2X7-P2Y1 inhibitory control significantly and increases the P2X3R-mediated nociceptive responses at the DRG level. Both the Western and behavioral analyses suggest that activation of p38 is necessary for the inhibitory control of P2X3Rs expression through the P2X7R-P2Y1R pathway. From these observations, we conclude that an activation of P2X7R-P2Y1R would lead to the activation of p38 and down-regulation of P2X3Rs, resulting in less P2X3Rs transported to the central and peripheral nerve terminals (Fig. 7).

## Discussion

Our studies show that activation of p38 in DRG neurons plays a critical role in the P2X7-P2Y1 inhibitory control of P2X3R expression in neurons (Figs. 1, 3). Since the disruption of SGC functions by the glia Krebs cycle inhibitor, FC, does not affect the inhibitory action of p38 on P2X3R expression (Fig. 1a), the observation suggests that the p38 in SGCs is not involved in the P2X7-P2Y1 control and the activation of neuronal p38 is sufficient to control the P2X3R expression in DRG neurons.

The p38 activator, Anis, is known to be a protein synthesis inhibitor [36]. It has been shown that Anis, at the dose (10 nM) used in our study, rarely affects the incorporation of [^3^H]lysine into protein in DRGs [36]. This together with our finding that Anis inhibits the P2X3R but not P2X7R expression (Fig. 1b) strongly supports our assertion that the Anis effect on P2X3R expression is through its activation of p38 activity, rather than an effect on protein synthesis. Pretreatment the JNK inhibitor, SP600125, did not alter the Anis effect on P2X3R confirming that JNKs are not involved in the action of Anis on P2X3R expression (Fig. 1c).

Since p38 activation can overcome the blocking action of P2X7Rs or P2Y1Rs on P2X3R expression (Fig. 5), this places p38 downstream of the P2X7-P2Y1 pathway (Fig. 7). In the presence of the p38 blocker, SB, P2X7R-P2Y1R can no longer exert an inhibitory control of P2X3R expression (Fig. 6), leading to the conclusion that p38 activation is necessary for the P2X7-P2Y1R inhibitory control of P2X3R expression to take place. Activation of p38 would result in a reduction in P2X3R expression in neuronal somata and thus less P2X3Rs transported to the central and peripheral nerve terminals (Fig. 7). This would reduce pain perception in the periphery or synaptic transmission in the spinal cord.

Under control conditions, pp38 was found in ~20 % of P2X3R-positive DRG neurons (Fig. 2) and P2X3R was present in ~40 % of the neurons (Fig. 6). Thus, it appears that only ~8 % of all DRG neurons or ~8/40 = ~20 % of P2X3R-containing neurons express pp38 and exert P2X7-P2Y1R inhibitory control of P2X3R expression. This is unlikely to be accurate because pp38 was found to be endogenously active under control conditions (Fig. 6b). As a result of pp38 inhibition of P2X3R expression in those cells, the number of pp38 and P2X3R double-labeled cells would be underestimated. Treatment of DRG with the p38 inhibitor, SB, resulted in a ~13 % increase in P2X3R neurons expressing p38 (Fig. 6b), suggesting that ~(8 + 13) = ~21 % of total neurons or ~21/(40 + 13) = ~40 % of P2X3R-containing neurons may express both P2X3R and pp38. This rough estimate illustrates the likelihood that a substantial percentage of P2X3R-containing cells exert pp38-dependent P2X7-P2Y1 inhibitory control of the P2X3R expression.

We observed an increase in P2X3R expression in complete Freund’s Adjuvant (CFA)-treated inflamed rats, even though the P2X7R expression is upregulated [15, 20]. Others showed that p38 is activated in DRG neurons after inflammation [41]. Thus, the enhancements in endogenous P2X7Rs and p38 are insufficient to block all of the increase in P2X3R expression induced by CFA. Additional activation of P2X7R was found to be required to block the CFA-induced P2X3R upregulation [10, 20].

In the periphery, injury-induced p38 activation mostly occurs in neurons [35, 42, 43]. Following tissue or nerve injury, mast cells, macrophages, neutrophils, keratinocytes and Schwann cells are recruited and/or activated at injured sites [44–46]. Inflammatory mediators, e.g., TNFα, nerve growth factor (NGF) and ATP are upregulated and released from those non-neuronal cells. They act directly on their receptors at sensory neurons, nerve fibers and their terminals [47–50] or indirectly through the release of prostanoids, e.g. PGE2 [51]. Initiation of mechanical allodynia induced by spinal nerve ligation has been attributed to TNFα-induced transient increase in pp38 in DRG neurons [42]. Exogenously-applied TNFα was found to activate p38 in cultured DRG neurons and increase TTX-resistant Na^+^ channel activity [43]. NGF was shown to activate p38 to potentiate TRPV1 responses after inflammation [41]. It has been shown that activation of P2Y1R in DRGs induces thermal sensitization or hyperalgesia [35, 52]. Kwon et al. [35] further found that activation of P2Y1Rs in peripheral nerve terminals by ATP released from damaged tissues after carrageenan-induced inflammation enhances pp38 expression in DRG neurons and results in TRPV1 upregulation.

There are fewer examples that p38-mediated changes in receptor or channel activity result in the reduction of hyperalgesia or chronic pain. Bhattacharjee’s group found that activation of p38 increased the activities of Na^+^ activated K^+^ channels thus reducing the activity of DRG neurons [53]. In our study, we showed that the activation of P2Y1Rs in neurons, induced by ATP released from activated P2X7R in SGCs, increases neuronal pp38 production (Fig. 3). Instead of causing an increase in the activity in DRG somata, this results in down-regulation of P2X3R expression and thus a reduction of nociceptor activity (Figs. 6, 7) [20].

Major differences exist between our results and those of Kwon et al. [35]. In addition to the opposite consequence between the excitatory P2Y1 → pp38 → TRPV1 cascade and inhibitory P2Y1 → pp38 → P2X3 cascade, the characteristics of the two responses are different. In the P2Y1 → pp38 → TRPV1 cascade, application of a P2Y1 agonist is required to stimulate the expression of TRPV1. Thermal hyperalgesia, but not mechanical allodynia, is affected by a P2Y1 antagonist [35]. In our previous study, applications of a P2Y1 agonist can alter mechanical allodynia induced by CFA (Figure S6 in [20]). It is of interest to determine the downstream events controlled by pp38 activation to produce pain-causing upregulation of TRPV1 versus analgesic down-regulation of P2X3Rs.

Since p38 activation was found mostly to enhance hyperalgesia, many proposed to use p38 inhibitors to treat chronic pain conditions [54]. There are at least two possible drawbacks to such an approach. One is that p38 is involved in many cell functions in addition to the induction of chronic pain [46, 54, 55]. Blocking p38 may also disrupt some essential cell functions and produce unexpected side effects. The second drawback is that such an approach not only blocks the pain-inducing effects, but also inhibits pain-reducing actions of p38 as we observed here. As we have shown before [4, 10, 20], P2X7R activation produces both excitatory and inhibitory action on P2X3R responses in DRGs, thus providing complex and interesting modulations of P2X3R activity. A better strategy is to aim at downstream targets of p38 which link directly to the production of specific chronic pain conditions.

## Methods

### Animals

Male Sprague–Dawley rats (250–350 g) were anesthetized with pentobarbital (50 mg/kg, i.p.) or with halothane (3 % for induction and 2 % for maintenance). L4 and L5 DRGs were used for experiments. All experimental procedures were approved by the Institutional Animal Care and Use Committee at University of Texas Medical Branch and were in accordance with the guidelines of the National Institutes of Health and of the International Association for the Study of Pain (IASP).

### Western blots

L4 and L5 DRGs were removed from normal rats and recovered in an oxygenated external solution (in mM) (130 NaCl, 5 KCl, 2 KH_2_PO_4_, 2.5 CaCl_2_, 1 MgCl_2_, 10 glucose, and 10 HEPES, pH7.4, osmolarity, 295–300 mosM) at room temperature for 30 min. DRGs were then treated with various drugs dissolved in the same solution for 1–3 h. DRGs were lysed in RIPA buffer (Thermal Scientific) containing protease inhibitor cocktail (Roche). For pp38 experiments, phosphatase Inhibitor III (Sigma) and phosphatase Inhibitor II (Sigma) were added into the RIPA buffer [56]. The cell lysates were centrifuged at 10,000×*g* for 10 min at 4 °C. Equal volumes of total protein sample from each group determined by BCA (Pierce) were loaded onto a SDS-PAGE, transferred to a PVDF membrane, incubated with a blocking buffer (1× TBS with 5 % w/v fat free dry milk) for 2 h, and then with rabbit anti-P2X3R (1:1000; Alomone) for 2 h at room temperature, or with mouse anti-pp38(1:1000; Cell Signaling) for 24 h at 4 °C. The PVDF membrane was then washed 5 times with TBST (1× TBS and 0.1 % Tween 20), incubated with Clean-Blot IP Detection Reagent (HRP) (1:1000; Thermo Scientific) for 1 h at room temperature and washed 5 times with TBST. For pp38 experiments, we used anti-mouse peroxidase-conjugated secondary antibody (1:1000, Chemicon). The immunoreactive proteins were detected by an enhanced chemiluminescence kit (Amersham Biosciences) and visualized by exposing the PVDF membrane onto an X-ray film. Loading controls were determined by re-probing the membrane with mouse anti-Actin (1:30,000, Chemicon) for 2 h or with rabbit anti-p38 (1:200; Santa Cruz) overnight at 4 °C for the pp38 experiment. After washing, the membrane was incubated with Blotting Grade Affinity Purified Goat Anti-Mouse IgG (H + L)-HRP Conjugate (1:15,000; Bio-Rad) for 1 h or with anti-rabbit peroxidase-conjugated secondary antibody (1:1000; Santa Cruz) for 1 h at room temperature. The intensity of protein bands was determined using the ImageJ software.

### Immunohistochemical procedure

To study the effect of a drug on the distribution of pp38 and/or P2X3Rs in DRG neurons, 10 µl of the drug at a proper concentration was applied intrathecally twice a day for 3 days. Rats were then deeply anesthetized with pentobarbital (80 mg/kg, i.p.) and transcardially perfused with normal saline (~50 ml) followed by a fixative (4 % paraformaldehyde plus 0.2 % picric acid in 0.1 M phosphate buffer, ~300 ml). L4 and L5 DRGs were then removed, put in a 30 % sucrose solution and stored at 4 °C overnight. Tissue was then embedded in OCT compound with liquid nitrogen and stored at −70 °C. Transverse sections of DRGs (10 µm thick) were cut on a cryostat and thaw-mounted onto glass slides. After drying, tissue sections were washed with PBS three times and incubated in PBS containing 10 % normal goat serum and 0.3 % Triton-100 for 1 h to block non-specific binding. The sections were then incubated with primary guinea pig anti-P2X3 receptor polyclonal antibody (Millipore, 1:5000) for 48 h or rabbit anti-pp38 monoclonal antibody (Cell Signaling, 1:500) for 36 h. After washing with PBS, the sections were incubated with secondary Alexa Fluor 594 conjugated goat anti-guinea pig IgG (Invitrogen, 1:200) or secondary Alexa Fluor 488 conjugated goat anti-rabbit IgG (Invitrogen, 1:200) for 1 h. Primary and secondary antibodies were diluted in PBS containing 1 % normal goat serum and 0.3 % Triton-100. To double stain the tissue with P2X3R and pp38, sections were stained first with P2X3 receptor antibody and stained subsequently with pp38 antibody using the procedures described before. All staining procedures were conducted at room temperature.

### Drug application

To block the activity of P2X7Rs, either the P2X7R antagonist oxATP (100 µM, Sigma) [57] or a highly selective P2X7R antagonist A740003 (250 nM, Sigma) was used. At 250 nM, A740003 inhibits >90 % of P2X7 receptor-mediated Ca^2+^ influx [58]. To activate P2Y1Rs, the agonist 2mesADP (100 µM, Sigma) was used. At 100 µM, 2mesADP was found to excite P2Y1 receptors in inhibitory neurons in the hippocampal slice [38]. To inhibit P2Y1R activity, the potent selective P2Y1R antagonist, MRS (60 µM, Tocris) was incubated with DRGs. In neocortical subventricular zone slices, MRS, at 50 µM, effectively blocks P2Y1R-mediated Ca^2+^ responses in intermediate neuronal progenitors [59]. To study the role of p38 in P2Y1R–P2X3R control, either the p38 inhibitor, SB (100 µM, Tocris) or the p38 activator, Anis (10 nM, Sigma) was used [60, 61]. The function of SGCs was disrupted by incubating DRGs in the glia Krebs cycle inhibitor, FC, (100 µM, Sigma) [62]. Unless stated otherwise, the incubation time for chemicals was 1 h.

### Behavioral experiments

All behavioral studies were performed under blind conditions. To inhibit the activity of p38, SB (100 µM, 10 µl) was applied intrathecally twice a day for 3 days. The behavioral experiment was started on the 4th day. Flinching of the rat left hindpaw in response to an intradermal paw injection of the P2X receptor agonist, α,β-meATP (50 nmole in 50 µl), was used to assess nociception elicited by activation of P2X3Rs [17, 63]. The nocifensive behavior was analyzed according to a previously described method [17, 40]. In response to α,β-meATP injection, rats not only lifted the injected paw more frequently, but also kept the paw in the air for a longer period. We therefore used paw withdrawal (PW) duration, i.e., the accumulative duration that the hindpaw was lifted in the air in a 1 min time bin, to assess flinching behaviors. Since PW duration depends on both paw lift frequency and duration, it gives a more accurate measure of nociception than flinching frequency, i.e., the number of paw lifts per min—a parameter commonly used to assess flinching behaviors.

### Data analyses

Data were expressed as mean ± SE. When the significance of changes between two groups was assessed, Student’s t test was used for data that were normally distributed. The Mann–Whitney Rank Sum Test was used when the normality test failed. When means from more than two groups were compared, one-way analysis of variance (ANOVA) followed by the Holm-Sidak post hoc test was used with normally distributed data sets. Kruskal–Wallis One Way Analysis of Variance on Ranks followed by Dunn’s Method was used when data were not normally distributed. A *P* < 0.05 was considered significant.
